# (*Z*)-3-(2-Meth­oxy­benz­yl)-1,5-benzo­thia­zepin-4(5*H*)-one

**DOI:** 10.1107/S160053681202661X

**Published:** 2012-06-16

**Authors:** R. Selvakumar, M. Bakthadoss, D. Lakshmanan, S. Murugavel

**Affiliations:** a Department of Organic Chemistry, University of Madras, Maraimalai Campus, Chennai 600 025, India; b Department of Physics, C. Abdul Hakeem College of Engineering & Technology, Melvisharam, Vellore 632 509, India; c Department of Physics, Thanthai Periyar Government Institute of Technology, Vellore 632 002, India

## Abstract

In the title compound, C_17_H_15_NO_2_S, the seven-membered thia­zepine ring adopts a distorted twist-boat conformation. The dihedral angle between the mean planes of the benzothia­zepin ring system and the attached benzene ring is 47.7 (1)°. In the crystal, pairs of N—H⋯O hydrogen bonds link inversion-related mol­ecules into dimers, generating *R*_2_^2^(8) ring motifs. These dimers are further connected into a chain along the *a* axis by C—H⋯O hydrogen bonds, resulting in *R*_2_^2^(14) ring motifs. The crystal packing also features C—H⋯π inter­actions.

## Related literature

For the pharmaceutical properties of thia­zepin derivatives, see: Tomascovic *et al.* (2000 ▶). For related structures, see: Sridevi *et al.* (2011 ▶); Sabari *et al.* 2011 ▶). For ring-puckering parameters, see: Cremer & Pople (1975 ▶). For hydrogen-bond motifs, see: Bernstein *et al.* (1995 ▶).
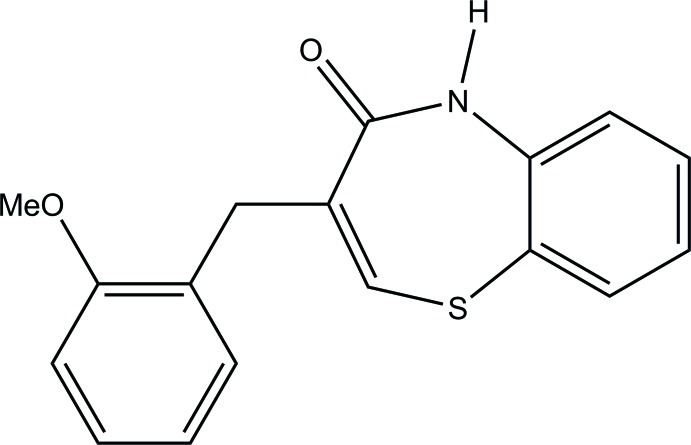


## Experimental

### 

#### Crystal data

C_17_H_15_NO_2_S
*M* *_r_* = 297.36
Triclinic, 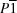

*a* = 8.6665 (5) Å
*b* = 9.7612 (4) Å
*c* = 10.1328 (5) Å
α = 108.181 (3)°
β = 101.561 (2)°
γ = 103.217 (3)°
*V* = 757.83 (7) Å^3^
*Z* = 2
Mo *K*α radiation
μ = 0.22 mm^−1^
*T* = 293 K
0.23 × 0.21 × 0.15 mm

#### Data collection

Bruker APEXII CCD diffractometer
Absorption correction: multi-scan (*SADABS*; Sheldrick, 1996 ▶) *T*_min_ = 0.951, *T*_max_ = 0.968
16048 measured reflections
4137 independent reflections
2797 reflections with *I* > 2σ(*I*)
*R*_int_ = 0.041

#### Refinement

*R*[*F* ^2^ > 2σ(*F* ^2^)] = 0.046
*wR*(*F* ^2^) = 0.146
*S* = 1.01
4137 reflections
191 parameters
H-atom parameters constrained
Δρ_max_ = 0.29 e Å^−3^
Δρ_min_ = −0.26 e Å^−3^

### 

Data collection: *APEX2* (Bruker, 2004 ▶); cell refinement: *SAINT* (Bruker, 2004 ▶); data reduction: *SAINT*; program(s) used to solve structure: *SHELXS97* (Sheldrick, 2008 ▶); program(s) used to refine structure: *SHELXL97* (Sheldrick, 2008 ▶); molecular graphics: *ORTEP-3* (Farrugia (1997 ▶); software used to prepare material for publication: *SHELXL97* and *PLATON* (Spek, 2009 ▶).

## Supplementary Material

Crystal structure: contains datablock(s) global, I. DOI: 10.1107/S160053681202661X/hb6847sup1.cif

Structure factors: contains datablock(s) I. DOI: 10.1107/S160053681202661X/hb6847Isup2.hkl

Supplementary material file. DOI: 10.1107/S160053681202661X/hb6847Isup3.cml

Additional supplementary materials:  crystallographic information; 3D view; checkCIF report

## Figures and Tables

**Table 1:** Hydrogen-bond geometry (Å, °) *Cg* is the centroid of the C11–C16 ring.

*D*—H⋯*A*	*D*—H	H⋯*A*	*D*⋯*A*	*D*—H⋯*A*
N1—H1⋯O1^i^	0.86	2.00	2.8545 (17)	171
C5—H5⋯O1^ii^	0.93	2.39	3.308 (2)	171
C3—H3⋯*Cg* ^iii^	0.93	2.69	3.432 (2)	138
C17—H17*C*⋯*Cg* ^iv^	0.96	2.90	3.664 (2)	137

Symmetry codes: (i) 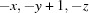; (ii) 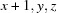; (iii) 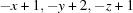; (iv) 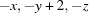.

**Fig. 1.**
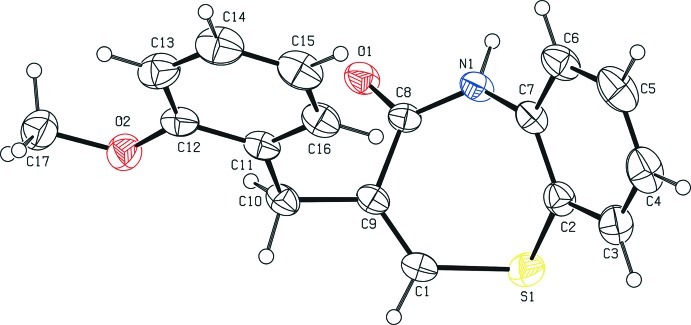
Molecular structure of the title compound showing displacement ellipsoids at the 30% probability level.

**Fig. 2.**
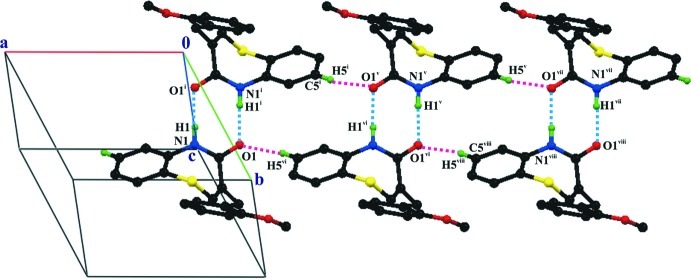
The crystal packing of the title compound, showing the chains along the a axis. H atoms not involved in the intermolecular interactions (dashed lines) have been omitted for clarity. [Symmetry code: (i) -x, -y, -z; (v) -1 - x, 1 - y, z; (vi) -1 + x, y, z; (vii) -2 - x, 1 - y, -z; (viii) -2 + x, y, z].

**Fig. 3.**
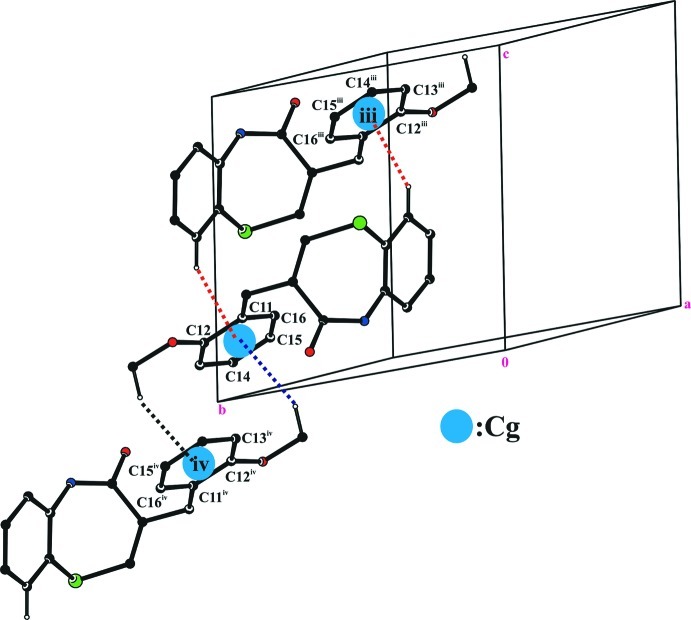
A view of the C—H···π interactions, in the crystal structure of the title compound. Cg is the centroid of the (C11–C16) benzene ring. [Symmetry code: (iii) 1 - x, 2 - y, 1 - z; (iv) -x, 2 - y, -z.]

## supplementary crystallographic information

### Comment

The title compound is used as an intermediate for the synthesis of dosulepin, which is an antidepressant of the tricyclic family. Dosulepin prevents reabsorbing of serotonin and noradrenaline in the brain, helps to prolong the mood lightening effect of any released noradrenaline and serotonin, thus relieving depression. The dibenzo[c,e]thiazepin derivatives exhibit chiroptical properties (Tomascovic *et al.,* 2000). As part of our studies in this area, we now describe the crystal structure of the title compound, (I).

Fig. 1. shows a displacement ellipsoid plot of (I), with the atom numbering scheme. The seven membered thiazepine ring (N1/S1/C1/C2/C7/C8/C9) adopts twist-boat conformation as indicated by puckering parameters (Cremer & Pople, 1975) QT = 1.0218 (14) Å, θ_2_ = 74.4 (1)°, φ_2_ = 178.1 (1)° and φ_3_ = 174.3 (4)°. The dihedral angle between the benzothiazepin ring system and the benzene ring is 47.7 (1)°. The geometric parameters of the title molecule agree well with those reported for similar structures (Sridevi *et al.,* 2011, Sabari *et al.,* 2011).

In the crystal, the molecules at *x, y, z* and *-x, 1-y, -z* are linked by N1—H1···O1 hydrogen bonds into cyclic centrosymmetric *R_2_^2^*(8) dimers (Bernstein *et al.,* 1995). These dimers are further connected into chains along the *a* axis *via* C5—H5···O1 hydrogen bonds (Table 1), resulting in *R_2_^2^*(14) ring motifs [Fig. 2; symmetry code as in Fig. 2].

The crystal packing also features two C—H···π interactions, the first one between a H3 atom and neighbouring benene ring (C11–C16), with a C3—H3···Cg^iii^ seperation of 2.69 Å and the second one between methyl H17C atom and neighbouring benzene ring (C11–C16), with a C17—H17C···Cg^iv^ seperation of 2.90 Å (Fig. 3 and Table 1; Cg is the centroid of the C11–C16 benzene ring, Symmetry code as in Fig.3).

### Experimental

A mixture of (*Z*)-methyl 2-(bromomethyl)-3-(2-methoxyphenyl)acrylate 2 mmol) and *o*-aminothiophenol (2 mmol) in the presence of potassium *tert*-butoxide (4.8 mmol) in dry THF (10 ml) was stirred at room temperature for 1 h. After the completion of the reaction as indicated by TLC, the reaction mixture was concentrated and the resulting crude mass was diluted with water (20 ml) and extracted with ethyl acetate (3 *x* 20 ml). The organic layer was washed with brine (2 *x* 20 ml) and dried over anhydrous sodium sulfate. The organic layer was concentrated, which successfully provide the crude final product ((*Z*)-3-(2-methoxybenzyl)benzo[*b*][1,4]thiazepin-4(5*H*)-one). The final product was purified by column chromatography on silica gel to afford the title compound in good yield (42%). Recrystallisation from ethyl acetate solution afforded colourless blocks.

### Refinement

H atoms were positioned geometrically, with C–H = 0.93–0.97 Å and constrained to ride on their parent atom, with *U*_iso_(H) =1.5*U*_eq_ for methyl H atoms and 1.2*U*_eq_(C) for other H atoms.

### Crystal data

**Table:**

C_17_H_15_NO_2_S	*Z* = 2
*M**_r_* = 297.36	*F*(000) = 312
Triclinic, *P*1	*D*_x_ = 1.303 Mg m^−^^3^
Hall symbol: -P 1	Mo *K*α radiation, λ = 0.71073 Å
*a* = 8.6665 (5) Å	Cell parameters from 4197 reflections
*b* = 9.7612 (4) Å	θ = 2.2–29.5°
*c* = 10.1328 (5) Å	µ = 0.22 mm^−^^1^
α = 108.181 (3)°	*T* = 293 K
β = 101.561 (2)°	Block, colourless
γ = 103.217 (3)°	0.23 × 0.21 × 0.15 mm
*V* = 757.83 (7) Å^3^	

### Data collection

**Table:**

Bruker APEXII CCD diffractometer	4137 independent reflections
Radiation source: fine-focus sealed tube	2797 reflections with *I* > 2σ(*I*)
Graphite monochromator	*R*_int_ = 0.041
Detector resolution: 10.0 pixels mm^-1^	θ_max_ = 29.5°, θ_min_ = 2.2°
ω scans	*h* = −10→11
Absorption correction: multi-scan (*SADABS*; Sheldrick, 1996)	*k* = −13→13
*T*_min_ = 0.951, *T*_max_ = 0.968	*l* = −13→14
16048 measured reflections	

### Refinement

**Table:**

Refinement on *F*^2^	Primary atom site location: structure-invariant direct methods
Least-squares matrix: full	Secondary atom site location: difference Fourier map
*R*[*F*^2^ > 2σ(*F*^2^)] = 0.046	Hydrogen site location: inferred from neighbouring sites
*wR*(*F*^2^) = 0.146	H-atom parameters constrained
*S* = 1.01	*w* = 1/[σ^2^(*F*_o_^2^) + (0.0717*P*)^2^ + 0.1349*P*] where *P* = (*F*_o_^2^ + 2*F*_c_^2^)/3
4137 reflections	(Δ/σ)_max_ < 0.001
191 parameters	Δρ_max_ = 0.29 e Å^−^^3^
0 restraints	Δρ_min_ = −0.26 e Å^−^^3^

### Special details

**Table:**

Geometry. All e.s.d.'s (except the e.s.d. in the dihedral angle between two l.s. planes) are estimated using the full covariance matrix. The cell e.s.d.'s are taken into account individually in the estimation of e.s.d.'s in distances, angles and torsion angles; correlations between e.s.d.'s in cell parameters are only used when they are defined by crystal symmetry. An approximate (isotropic) treatment of cell e.s.d.'s is used for estimating e.s.d.'s involving l.s. planes.
Refinement. Refinement of *F*^2^ against ALL reflections. The weighted *R*-factor *wR* and goodness of fit *S* are based on *F*^2^, conventional *R*-factors *R* are based on *F*, with *F* set to zero for negative *F*^2^. The threshold expression of *F*^2^ > σ(*F*^2^) is used only for calculating *R*-factors(gt) *etc*. and is not relevant to the choice of reflections for refinement. *R*-factors based on *F*^2^ are statistically about twice as large as those based on *F*, and *R*- factors based on ALL data will be even larger.

### Fractional atomic coordinates and isotropic or equivalent isotropic displacement parameters (Å^2^)

**Table:**

	*x*	*y*	*z*	*U*_iso_*/*U*_eq_	
C1	0.1759 (2)	0.7930 (2)	0.46956 (18)	0.0507 (4)	
H1A	0.1877	0.8740	0.5533	0.061*	
C2	0.3850 (2)	0.64173 (18)	0.39653 (19)	0.0488 (4)	
C3	0.5478 (2)	0.6617 (2)	0.4686 (2)	0.0608 (5)	
H3	0.5776	0.6775	0.5668	0.073*	
C4	0.6642 (2)	0.6582 (2)	0.3954 (3)	0.0722 (6)	
H4	0.7726	0.6702	0.4437	0.087*	
C5	0.6218 (2)	0.6371 (3)	0.2510 (3)	0.0750 (7)	
H5	0.7016	0.6348	0.2018	0.090*	
C6	0.4611 (2)	0.6192 (2)	0.1781 (2)	0.0599 (5)	
H6	0.4332	0.6057	0.0803	0.072*	
C7	0.34186 (19)	0.62133 (17)	0.25091 (18)	0.0451 (4)	
C8	0.07211 (19)	0.67234 (17)	0.20182 (16)	0.0411 (3)	
C9	0.11639 (19)	0.80262 (17)	0.34308 (17)	0.0424 (4)	
C10	0.0753 (2)	0.93974 (18)	0.33034 (18)	0.0486 (4)	
H10A	0.0944	1.0126	0.4271	0.058*	
H10B	−0.0416	0.9100	0.2793	0.058*	
C11	0.1745 (2)	1.01587 (18)	0.25198 (17)	0.0470 (4)	
C12	0.1171 (2)	1.11473 (19)	0.19637 (17)	0.0513 (4)	
C13	0.2044 (3)	1.1847 (2)	0.1219 (2)	0.0668 (5)	
H13	0.1647	1.2489	0.0831	0.080*	
C14	0.3502 (3)	1.1584 (3)	0.1058 (2)	0.0756 (6)	
H14	0.4084	1.2051	0.0556	0.091*	
C15	0.4107 (3)	1.0648 (3)	0.1623 (2)	0.0704 (6)	
H15	0.5102	1.0490	0.1521	0.085*	
C16	0.3221 (2)	0.9936 (2)	0.2350 (2)	0.0568 (5)	
H16	0.3629	0.9294	0.2732	0.068*	
C17	−0.0912 (3)	1.2357 (2)	0.1686 (2)	0.0704 (6)	
H17A	−0.0129	1.3361	0.2124	0.106*	
H17B	−0.1929	1.2378	0.1917	0.106*	
H17C	−0.1121	1.2002	0.0650	0.106*	
N1	0.17774 (16)	0.59507 (15)	0.17075 (14)	0.0454 (3)	
H1	0.1400	0.5180	0.0899	0.055*	
O1	−0.06363 (14)	0.63859 (14)	0.11211 (12)	0.0530 (3)	
O2	−0.02545 (17)	1.13659 (15)	0.22258 (14)	0.0595 (3)	
S1	0.23445 (6)	0.63866 (6)	0.49135 (5)	0.05956 (17)	

### Atomic displacement parameters (Å^2^)

**Table:**

	*U*^11^	*U*^22^	*U*^33^	*U*^12^	*U*^13^	*U*^23^
C1	0.0560 (10)	0.0537 (9)	0.0413 (8)	0.0174 (8)	0.0217 (7)	0.0116 (7)
C2	0.0453 (9)	0.0419 (8)	0.0522 (9)	0.0133 (7)	0.0104 (7)	0.0113 (7)
C3	0.0508 (10)	0.0505 (10)	0.0644 (11)	0.0137 (8)	0.0004 (9)	0.0117 (8)
C4	0.0417 (10)	0.0606 (12)	0.0915 (16)	0.0142 (9)	0.0038 (10)	0.0098 (11)
C5	0.0451 (11)	0.0719 (13)	0.1002 (18)	0.0195 (9)	0.0327 (11)	0.0139 (12)
C6	0.0474 (10)	0.0650 (11)	0.0615 (11)	0.0185 (8)	0.0232 (8)	0.0108 (9)
C7	0.0377 (8)	0.0405 (8)	0.0489 (9)	0.0114 (6)	0.0122 (7)	0.0070 (6)
C8	0.0380 (8)	0.0453 (8)	0.0398 (7)	0.0105 (6)	0.0167 (6)	0.0141 (6)
C9	0.0404 (8)	0.0444 (8)	0.0435 (8)	0.0125 (6)	0.0207 (7)	0.0130 (6)
C10	0.0539 (10)	0.0462 (8)	0.0507 (9)	0.0177 (7)	0.0272 (8)	0.0158 (7)
C11	0.0503 (9)	0.0412 (8)	0.0405 (8)	0.0065 (7)	0.0170 (7)	0.0069 (6)
C12	0.0579 (10)	0.0475 (9)	0.0393 (8)	0.0071 (8)	0.0151 (7)	0.0103 (7)
C13	0.0782 (14)	0.0627 (12)	0.0582 (11)	0.0106 (10)	0.0229 (10)	0.0274 (9)
C14	0.0785 (15)	0.0759 (14)	0.0739 (14)	0.0068 (11)	0.0413 (12)	0.0306 (11)
C15	0.0611 (12)	0.0689 (13)	0.0770 (14)	0.0082 (10)	0.0382 (11)	0.0193 (11)
C16	0.0556 (10)	0.0518 (9)	0.0580 (10)	0.0113 (8)	0.0238 (9)	0.0134 (8)
C17	0.0779 (14)	0.0669 (12)	0.0699 (13)	0.0253 (11)	0.0168 (11)	0.0314 (10)
N1	0.0398 (7)	0.0468 (7)	0.0403 (7)	0.0128 (6)	0.0114 (5)	0.0047 (5)
O1	0.0427 (6)	0.0618 (7)	0.0467 (6)	0.0197 (5)	0.0108 (5)	0.0092 (5)
O2	0.0643 (8)	0.0612 (8)	0.0620 (8)	0.0232 (6)	0.0230 (6)	0.0295 (6)
S1	0.0660 (3)	0.0692 (3)	0.0533 (3)	0.0255 (2)	0.0227 (2)	0.0295 (2)

### Geometric parameters (Å, º)

**Table:**

C1—C9	1.323 (2)	C10—C11	1.507 (2)
C1—S1	1.7571 (19)	C10—H10A	0.9700
C1—H1A	0.9300	C10—H10B	0.9700
C2—C7	1.385 (2)	C11—C16	1.381 (3)
C2—C3	1.391 (3)	C11—C12	1.394 (3)
C2—S1	1.7687 (18)	C12—O2	1.364 (2)
C3—C4	1.367 (3)	C12—C13	1.386 (3)
C3—H3	0.9300	C13—C14	1.376 (3)
C4—C5	1.371 (3)	C13—H13	0.9300
C4—H4	0.9300	C14—C15	1.365 (4)
C5—C6	1.382 (3)	C14—H14	0.9300
C5—H5	0.9300	C15—C16	1.385 (3)
C6—C7	1.386 (2)	C15—H15	0.9300
C6—H6	0.9300	C16—H16	0.9300
C7—N1	1.410 (2)	C17—O2	1.421 (2)
C8—O1	1.2353 (19)	C17—H17A	0.9600
C8—N1	1.338 (2)	C17—H17B	0.9600
C8—C9	1.495 (2)	C17—H17C	0.9600
C9—C10	1.497 (2)	N1—H1	0.8600
			
C9—C1—S1	124.92 (13)	C11—C10—H10B	108.8
C9—C1—H1A	117.5	H10A—C10—H10B	107.7
S1—C1—H1A	117.5	C16—C11—C12	118.41 (16)
C7—C2—C3	119.73 (17)	C16—C11—C10	122.51 (17)
C7—C2—S1	120.79 (13)	C12—C11—C10	119.07 (15)
C3—C2—S1	119.47 (15)	O2—C12—C13	124.34 (18)
C4—C3—C2	120.2 (2)	O2—C12—C11	115.28 (15)
C4—C3—H3	119.9	C13—C12—C11	120.37 (18)
C2—C3—H3	119.9	C14—C13—C12	119.5 (2)
C3—C4—C5	120.27 (18)	C14—C13—H13	120.2
C3—C4—H4	119.9	C12—C13—H13	120.2
C5—C4—H4	119.9	C15—C14—C13	121.1 (2)
C4—C5—C6	120.4 (2)	C15—C14—H14	119.4
C4—C5—H5	119.8	C13—C14—H14	119.4
C6—C5—H5	119.8	C14—C15—C16	119.2 (2)
C5—C6—C7	119.9 (2)	C14—C15—H15	120.4
C5—C6—H6	120.1	C16—C15—H15	120.4
C7—C6—H6	120.1	C11—C16—C15	121.4 (2)
C2—C7—C6	119.57 (16)	C11—C16—H16	119.3
C2—C7—N1	122.33 (15)	C15—C16—H16	119.3
C6—C7—N1	118.03 (15)	O2—C17—H17A	109.5
O1—C8—N1	120.15 (13)	O2—C17—H17B	109.5
O1—C8—C9	118.65 (14)	H17A—C17—H17B	109.5
N1—C8—C9	121.18 (14)	O2—C17—H17C	109.5
C1—C9—C8	122.35 (15)	H17A—C17—H17C	109.5
C1—C9—C10	122.86 (14)	H17B—C17—H17C	109.5
C8—C9—C10	114.62 (14)	C8—N1—C7	129.58 (13)
C9—C10—C11	113.97 (14)	C8—N1—H1	115.2
C9—C10—H10A	108.8	C7—N1—H1	115.2
C11—C10—H10A	108.8	C12—O2—C17	118.25 (15)
C9—C10—H10B	108.8	C1—S1—C2	98.07 (8)
			
C7—C2—C3—C4	−1.3 (3)	C16—C11—C12—O2	−177.21 (14)
S1—C2—C3—C4	177.35 (15)	C10—C11—C12—O2	1.7 (2)
C2—C3—C4—C5	0.9 (3)	C16—C11—C12—C13	2.0 (2)
C3—C4—C5—C6	0.0 (3)	C10—C11—C12—C13	−179.04 (16)
C4—C5—C6—C7	−0.5 (3)	O2—C12—C13—C14	177.81 (17)
C3—C2—C7—C6	0.8 (3)	C11—C12—C13—C14	−1.4 (3)
S1—C2—C7—C6	−177.84 (13)	C12—C13—C14—C15	−0.2 (3)
C3—C2—C7—N1	177.65 (15)	C13—C14—C15—C16	1.0 (3)
S1—C2—C7—N1	−1.0 (2)	C12—C11—C16—C15	−1.2 (3)
C5—C6—C7—C2	0.1 (3)	C10—C11—C16—C15	179.90 (17)
C5—C6—C7—N1	−176.89 (17)	C14—C15—C16—C11	−0.3 (3)
S1—C1—C9—C8	7.8 (2)	O1—C8—N1—C7	174.44 (16)
S1—C1—C9—C10	−177.20 (12)	C9—C8—N1—C7	−4.2 (3)
O1—C8—C9—C1	131.77 (18)	C2—C7—N1—C8	50.9 (3)
N1—C8—C9—C1	−49.6 (2)	C6—C7—N1—C8	−132.22 (18)
O1—C8—C9—C10	−43.6 (2)	C13—C12—O2—C17	1.0 (3)
N1—C8—C9—C10	135.01 (16)	C11—C12—O2—C17	−179.81 (15)
C1—C9—C10—C11	118.63 (18)	C9—C1—S1—C2	58.22 (17)
C8—C9—C10—C11	−66.03 (18)	C7—C2—S1—C1	−60.82 (15)
C9—C10—C11—C16	−19.6 (2)	C3—C2—S1—C1	120.55 (15)
C9—C10—C11—C12	161.57 (14)		

### Hydrogen-bond geometry (Å, º)

Cg is the centroid of the C11–C16 ring.

**Table:**

*D*—H···*A*	*D*—H	H···*A*	*D*···*A*	*D*—H···*A*
N1—H1···O1^i^	0.86	2.00	2.8545 (17)	171
C5—H5···O1^ii^	0.93	2.39	3.308 (2)	171
C3—H3···*Cg*^iii^	0.93	2.69	3.432 (2)	138
C17—H17*C*···*Cg*^iv^	0.96	2.90	3.664 (2)	137

Symmetry codes: (i) −*x*, −*y*+1, −*z*; (ii) *x*+1, *y*, *z*; (iii) −*x*+1, −*y*+2, −*z*+1; (iv) −*x*, −*y*+2, −*z*.
